# Evaluating the Divide Between Patients’ and Physicians’ Perceptions of Adult-Onset Still’s Disease Cases: Insights from the PRO-AOSD Survey

**DOI:** 10.3390/jcm14197034

**Published:** 2025-10-04

**Authors:** Norbert Blank, Ioana Andreica, Jürgen Rech, Zekayi Sözen, Eugen Feist

**Affiliations:** 1 Department of Internal Medicine V, Division of Rheumatology, University Hospital Heidelberg, 69120 Heidelberg, Germany; norbert.blank@med.uni-heidelberg.de; 2 Center for Rare Diseases Heidelberg (ZSEHD), University Hospital Heidelberg, 69120 Heidelberg, Germany; 3 Rheumatology Research, Faculty of Medicine, Ruhr-Universität Bochum, 44780 Bochum, Germany; ioana.andreica@elisabethgruppe.de; 4 Internal Medicine and Rheumatology, Rheumazentrum Ruhrgebiet, 44649 Herne, Germany; 5 Department of Internal Medicine 3—Rheumatology and Immunology, Friedrich-Alexander University (FAU) Erlangen-Nürnberg and Universitätsklinikum Erlangen, 91054 Erlangen, Germany; juergen.rech@uk-erlangen.de; 6 Deutsches Zentrum für Immuntherapie (DZI), Friedrich-Alexander-Universität Erlangen-Nürnberg and Uniklinikum Erlangen, 91054 Erlangen, Germany; 7 Center for Rare Diseases Erlangen (ZSEER), Friedrich-Alexander-Universität Erlangen-Nürnberg and Uniklinikum Erlangen, 91054 Erlangen, Germany; 8 Novartis Pharma AG, CH-4056 Basel, Switzerland; zek_soez@hotmail.com; 9 Department of Rheumatology and Clinical Immunology, Helios Fachklinik Vogelsang-Gommern Klinik für Rheumatologie, 39245 Gommern, Germany; 10 Experimental Rheumatology, Otto-von-Guericke Universität Magdeburg, 39106 Magdeburg, Germany

**Keywords:** rheumatology, Still’s disease, Adult-Onset, inflammation, patient-reported outcome measures

## Abstract

**Background/Objectives**: This study aims to report and compare data from the PRO-AOSD (patient-reported outcomes adult-onset Still’s disease) survey in patients with AOSD from the perspective of patients and their treating physicians. **Methods**: PRO-AOSD comprised blinded patient and physician surveys. The surveys were designed to assess perceived symptoms and physical impairment. Outcomes reported here include patient demographics; physicians’ assessment of the patient’s health state; physician-reported laboratory findings; pain; disease activity; symptoms; physicians’ treatment goals; and the impact of lifestyle factors on disease improvement. **Results**: Adult patients with AOSD were recruited from 19 centers in Germany. A total of 124 patients were included, with 74 (59.7%) females; the mean age was 45.5 years. The mean time from first symptom to diagnosis was 2 years, and the mean time was 7 years from diagnosis to survey completion (N = 123). Of 107 patients, most (81.3%) had inactive disease defined by CRP levels. At the time of the survey, around two-thirds of patients were receiving biologic therapy, with 84.1% (69/82) reporting an improvement in symptoms. Many patients had previously received antibiotics (47.6% [*n* = 58] and 30.4% [*n* = 37], per patient and physician reports, respectively). Persistent symptoms were reported more frequently by patients than by physicians, including back pain (39.5%), fatigue and weakness (38.7%), and joint inflammation (27.4%). Physicians classified 35.5% of patients as symptom-free. Patients reported exercise as having a positive impact on symptoms (52.4%), while stress (74.2%) and sleep deprivation (62.9%) were reported to worsen symptoms. Targeting systemic manifestations, such as the absence of fever (58.1%, *n* = 72), was considered the most important treatment goal by physicians. **Conclusions**: Data from PRO-AOSD highlight distinct differences between patients’ and physicians’ interpretations of the same cases of AOSD. **Prior Presentation**: These data were presented at the German Congress of Rheumatology (DGRh; 30 August–2 September 2023; Leipzig, Germany).

## 1. Introduction

Still’s disease is a rare auto-inflammatory disease (AID) of unknown etiology, comprising a continuum from the juvenile form (systemic juvenile idiopathic arthritis [sJIA]) to the adult form (adult-onset Still’s disease [AOSD]) [1,2]. It is characterized by high-spiking fever, arthritis, an evanescent salmon-colored skin rash, and pharyngitis [3,4,5,6]. Common laboratory manifestations include elevated levels of ferritin, C-reactive protein (CRP), erythrocyte sedimentation rate (ESR), neutrophils, leukocytes, and platelets [4]. CRP is a well-known biomarker with a high sensitivity for disease activity monitoring [7].

AOSD is typically classified based on the type of symptoms experienced and/or the disease pattern over time. Symptoms can manifest in two ways: with patients experiencing systemic features (such as high fever, skin rash, and, potentially, internal organ involvement) or with patients exhibiting articular manifestations (like joint pain and arthritis) [4,6,8]. The disease can follow three patterns: monophasic, characterized by a single episode of the disease lasting weeks to months but resolving within a year; polyphasic, marked by multiple intermittent episodes alternating with periods of remission; or chronic, involving continuous symptoms and persistent disease activity [6,8,9].

There are only a few published treatment guidelines for AOSD; however, the typical treatment paradigm is changing from glucocorticoid therapy to a steroid-sparing treatment, especially with biologics, initiated as early as possible following diagnosis [4,5,6,9,10,11].

Although it has been established that health-related quality of life (HRQoL) is reduced in patients with rheumatic diseases [12,13], few studies have assessed HRQoL outcomes in AOSD to date. One recent study in patients with AOSD showed a 25% reduction in HRQoL when compared with age- and gender-matched healthy controls [14]. The relevance of patient-reported outcomes (PROs) is being increasingly emphasized, as they provide multi-dimensional information on a patient’s disease, including insights into which outcomes are most important to patients [13,14]. Studies in rheumatoid arthritis have shown that PROs can show a greater sensitivity to treatment effects when compared with physician-reported outcomes [15,16,17]. The literature on PROs versus physician-reported outcomes in AOSD is limited.

PRO-AOSD is a survey of disease-related symptoms and physical impairments, HRQoL, work productivity, and lifestyle factors of patients with AOSD in Germany. As there are no AOSD-specific PRO measures, PRO-AOSD was designed to compare patient and physician perspectives while incorporating validated tools, such as the Work Productivity and Activity Impairment (WPAI) questionnaire, Short Form-36 (SF-36), Pouchot Score, and Disease Activity Score 28 (DAS28). Here, we report patients’ perceptions of their current symptoms and impairment and compare them with the assessments of their treating physicians.

## 2. Materials and Methods

### 2.1. Study Design and Patients

Patients were recruited from 19 centers in Germany to participate in the PRO-AOSD survey. Adult patients (aged ≥ 18 years) with a confirmed diagnosis of AOSD were identified by their attending physician as being suitable candidates. Two surveys were used for PRO-AOSD: one was a patient survey, completed by patients with AOSD using electronic tablets on the day of their appointment, while the other was a physician survey, conducted online via personalized, coded links following the patient’s appointment (Supplementary Materials). Patients and physicians were blinded to each other’s survey responses, and all data were collected anonymously.

### 2.2. Assessments

The surveys were designed to assess perceived symptoms and physical impairment. The first part of the patient survey comprised 5 questions regarding demographic data, while the second part comprised 43 questions about symptoms and physical impairments associated with AOSD-related symptoms. The physician survey consisted of 27 questions relating to the patient’s AOSD symptoms and physical impairments (Supplementary Materials). This dual approach was taken to offer a comprehensive view of the disease, complementing patients’ perspectives with physicians’ assessments to reveal new insights for disease management. To facilitate comparison with other rheumatic diseases, validated tools (WPAI, SF-36, Pouchot Score, and DAS28) were included in both surveys.

Outcomes reported here include patient demographics (sex, age, weight, height, body mass index [BMI], current smoker, time to diagnosis, disease course, and current and past medications received); physicians’ assessments of the patient’s health state; laboratory findings as reported by physicians; pain; disease activity; symptoms; physicians’ goals when treating patients; and the impact of lifestyle factors on disease improvement. An additional analysis was performed to determine disease activity according to CRP levels; inactive, mild, and moderate-to-severe disease activity were defined as CRP levels < 10 mg/L, 10–30 mg/L, and >30 mg/L, respectively.

### 2.3. Statistical Analysis

For some questions, the number of responses deviated from the total sample size due to missing or implausible data. In these cases, the deviating sample size is indicated. McNemar’s test was used to compare physician reports of disease course versus patients’ own assessment of health (compared with the previous year) and patient-reported versus physician-reported symptoms. Correlations between patients’ and physicians’ interpretations of disease activity and pain were assessed using Pearson’s and Spearman’s rank correlation coefficients. Bivariate normality was evaluated using the Shapiro–Wilk test. Disease activity between groups, based on CRP level, was compared using Welch’s *t*-test. A *p*-value of <0.05 was considered statistically significant. All analyses were conducted using JASP software, with the latest version available at the time of each analysis (versions: 0.17.1; 0.17.2.1; 0.18.1 and 0.18.3).

### 2.4. Patient and Public Involvement

Patients and/or the public were not involved in the development of the research question, the design of this study, or the recruitment for and supervision of the study. Written informed consent was obtained from all patients before the study. 

### 2.5. Ethical Approval

The study was approved by the ethics committee of the Otto-von-Guericke University, Medical Faculty and University Hospital, Magdeburg (99/20; date of approval 21 July 2020); all participating institutions were covered by this ethical approval.

## 3. Results

### 3.1. Patient Demographics

The PRO-AOSD study included data from 124 adult patients with confirmed AOSD. The first patient completed the survey on 8 March 2021, and the last patient completed it on 14 November 2022. Out of 124 patients, 74 (59.7%) were female and 50 (40.3%) were male; the mean age of the entire study cohort was 45.5 years (SD 14.7; Table 1). The mean BMI of the study cohort was 27.0 kg/m^2^ (SD 5.8), and a total of 18 patients (14.5%) were smokers (Table 1).

#### 3.1.1. Time to Diagnosis

Patients experienced symptoms for a mean of 2 years prior to receiving a diagnosis of AOSD; the mean age (N = 123) at the onset of symptoms was 36 years, and at diagnosis, it was 38 years. At the time of the survey, it had been a mean of 9 years since patients experienced their first symptom (mean of 7 years since diagnosis) (N = 123). Out of 124 patients, a total of 117 (94.4%) patients were diagnosed by a rheumatologist or physician in the hospital/clinic, and 7 patients were diagnosed by other physicians, including dermatologists or general practitioners.

#### 3.1.2. Disease Course

Out of 124 patients, 28 (22.6%) were reported as having monophasic disease by physicians, and 53 (42.7%) were reported as having polyphasic/multiphasic disease, while 46 (37.1%) physicians reported chronic disease in their patients (physicians could select ≥1 disease classification per patient). Compared with the previous year, 42 (33.9%) patients reported similar health status, 44 (35.5%) patients reported an improvement (either ‘a little better’ or ‘much better’) in their health, and 38 (30.6%) patients reported a worsening (either ‘a little worse’ or ‘much worse’) of their health status. While 25 (80.6%) patients described their health status as ‘much worse’ than the previous year, a total of 101 (81.5%) patients were reported by their treating physician as having an improved global health status over the course of their treatment. For patients who experienced an improvement in their health status, all treating physicians also shared this perception.

#### 3.1.3. Current and Past Medication

**Figure 1:**
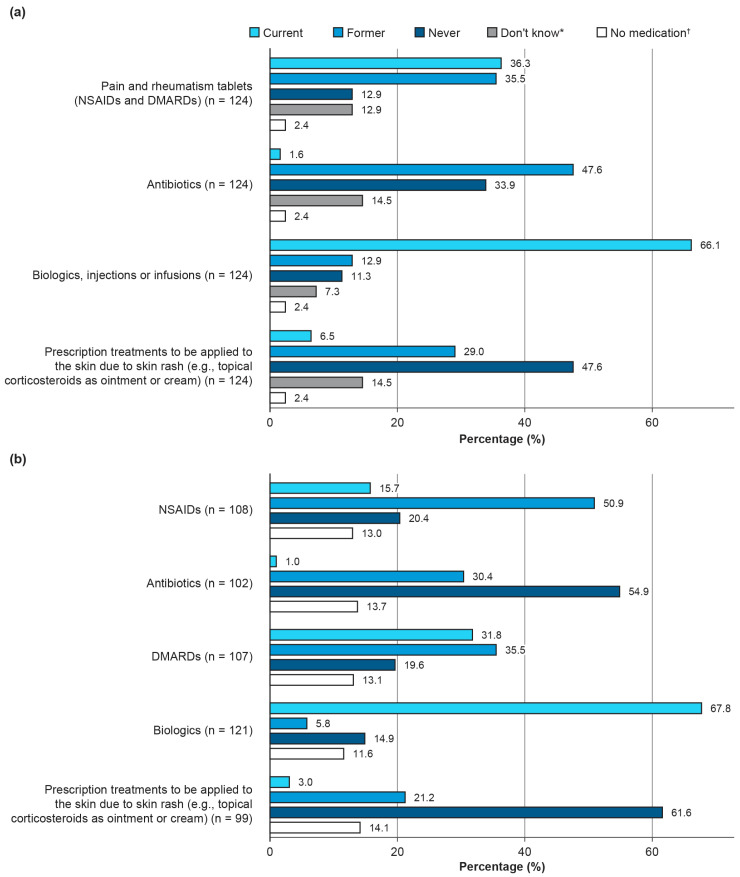
Current and past medications, as reported by (**a**) patients and (**b**) physicians. Question as it appeared on the patient survey: “What medications are you taking or have you previously because of the AOSD taken or administered?” Question as it appeared on the physician survey: “Which drug group is prescribed to patients?”. * Only requested from patients. ^†^ This refers to the patient not receiving any previous or current medication for the treatment of AOSD. AOSD, adult-onset Still’s disease; DMARD, disease-modifying anti-rheumatic drug; NSAID, non-steroidal anti-inflammatory drug.

Overall, 121 (97.6%) patients reported that they had received or were currently receiving medication to treat their condition, with 101 (83.5%) patients feeling that their symptoms had been sufficiently improved by the medication they were receiving (N = 121). At the time of the survey, around two-thirds of patients were receiving biologic therapy, reported by 82 of 124 (66.1%) patients and 82 of 121 (67.8%) physicians (Figure 1a,b). Of these patients, 69 of 82 (84.1%) reported an improvement in symptoms. Many patients had previously received antibiotics: 47.6% (*n* = 58) and 30.4% (*n* = 37) patients, per patient and physician reports, respectively (Figure 1a,b).

### 3.2. Laboratory Findings

The median results for all laboratory tests were within the normal range. The median serum ferritin level (*n* = 88) was 118.5 µg/L, the CRP level (*n* = 107) was 2.5 mg/L, ESR (*n* = 77) was 11.0 mm/h, the AST level (*n* = 87) was 27.0 IU/L, the ALT level (*n* = 107) was 25.0 IU/L, and the median leukocyte count (*n* = 89) was 6.3 Gpt/L, respectively (Table 2). A total of 12 (11.2%) patients with valid CRP levels (N = 107) had levels ≥2 times the upper limit of normal (≥20 mg/L).

### 3.3. Physicians’ Treatment Goals

**Figure 2:**
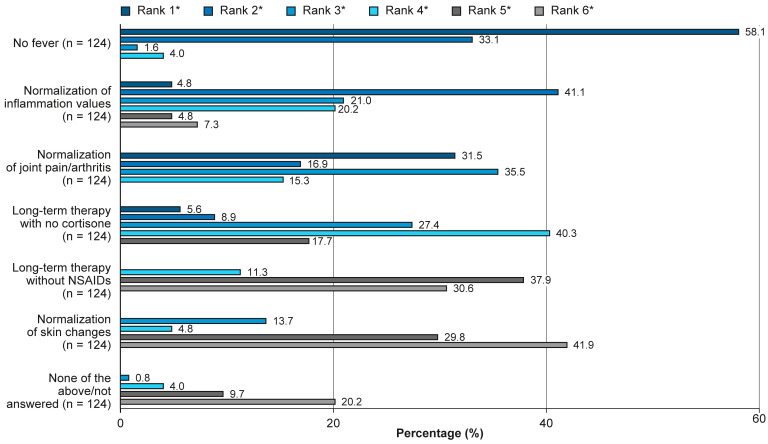
Therapy goals ranked by importance, as reported by physicians. Question as it appeared on the physician survey: “Which therapy goals are important to you?”. * Physicians ranked therapy goals from most important (Rank 1) to least important (Rank 6). NSAID, non-steroidal anti-inflammatory drug.

The most important treatment goal for physicians when treating patients with AOSD was targeting systemic manifestations (Figure 2). No fever had the highest priority (58.1%, *n* = 72), followed by normalization of joint pain/arthritis (31.5%, *n* = 39), long-term therapy with no cortisone (5.6%, *n* = 7), and normalization of inflammation values (4.8%, *n* = 6). Normalization of skin changes was not ranked as a priority by the responding physicians (Figure 2).

### 3.4. Physicians’ Assessments of Patients’ Health States

Physicians reported an improvement in global health state for 101 (81.5%) of their patients. Out of the 82 patients receiving biologic therapy at the time of the survey, 70 (85.4%) were reported as having an improved global health state by physicians. A total of 44 (35.5%) patients were classified as symptom-free, of whom 30 (68.2%) received a biologic agent.

### 3.5. Pain, Disease Activity, and Symptoms

The mean (SD) patient score for current pain was 2.5 (2.4) and 2.1 (2.4), as reported by patients and physicians, respectively. A total of 34 (27.4%) patients reported that they were experiencing no pain. There was a significant correlation between the patient and physician assessment of current pain (*p* < 0.001).

Patients reported a mean (SD) score of 2.7 (2.6) for disease activity and 2.6 (2.5) for current symptoms, while physicians reported a mean (SD) score of 2.0 (2.4) for assessment of current global disease activity. Patients rated their current disease activity as significantly higher than their physicians’ assessments (*p* < 0.001).

**Figure 3:**
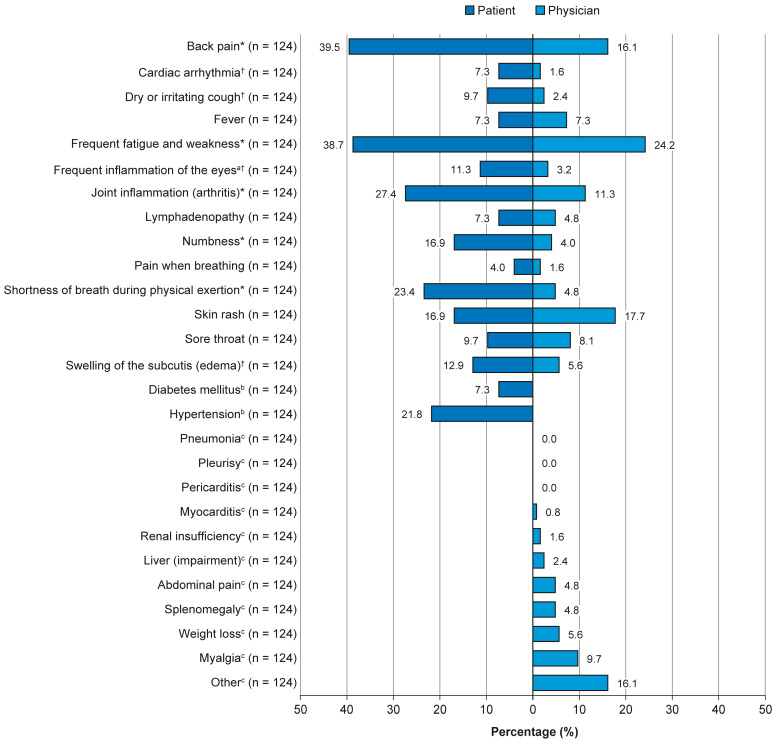
Current symptoms as reported by patients and physicians. Question as it appeared on the patient survey: “What symptoms do you suffer from today?” Question as it appeared on the physician survey: “What accompanying symptoms do the patient currently have? (Multiple answers possible)”. * *p* < 0.001; ^†^ *p* < 0.01; ^a^ accompanied by sensitivity to light and pain; ^b^ only requested from patients; ^c^ only requested from physicians.

Of the symptoms reported by both patients and physicians, 9 of 14 symptoms were reported significantly more frequently by patients (*p* < 0.01; Figure 3). The other symptoms showed no significant difference in reporting frequency between groups, and no symptoms were reported significantly more frequently by physicians. Hypertension was reported by 27 (21.8%) patients, while diabetes mellitus was reported by 9 (7.3%) patients (Figure 3). A total of 18 (14.5%) patients reported swollen joints; however, 65 (52.4%) patients reported that their joints were painful. Physicians reported that 14 (11.3%) patients had developed macrophage activation syndrome in the past.

### 3.6. Disease Activity Determined by CRP Levels

**Figure 4:**
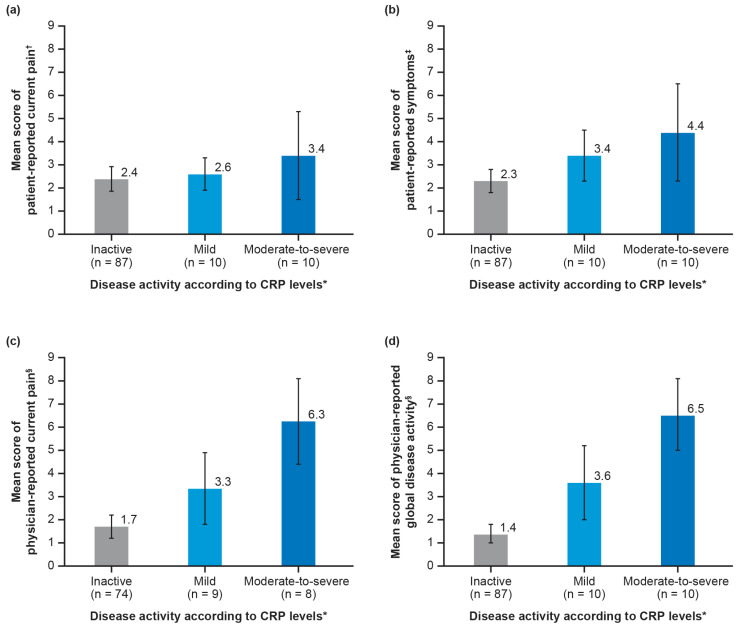
Mean score of patient- and physician-reported pain, global disease activity, and symptoms according to disease activity as measured by CRP levels*: (**a**) patient-reported current pain; (**b**) patient-reported symptoms; (**c**) physician-reported current pain; and (**d**) physician-reported global disease activity. Error bars represent a 95% confidence interval. * Disease activity was defined as (i) inactive: CRP < 10 mg/L; (ii) mild: CRP = 10–30 mg/L; and (iii) moderate to severe: >30 mg/L. ^†^ *p* < 0.596; ^‡^ *p* = 0.090; ^§^ *p* < 0.001. CRP, C-reactive protein.

Of 107 patients, 87 (81.3%) had inactive disease based on their CRP levels, 10 (9.3%) were classified as having mild disease activity, and 10 (9.3%) had moderate-to-severe disease activity. In patients with moderate-to-severe disease activity versus patients with inactive disease activity, a tendency towards higher patient-reported pain (*p* = 0.596) and symptoms (*p* = 0.090) was seen (Figure 4a,b). There were significant correlations between CRP-defined disease activity and the physician’s assessment of current pain or disease activity (both *p* < 0.001; Figure 4c,d).

### 3.7. Impact of External Factors on Disease Improvement

The impact of external factors on disease improvement is presented in Table 3. Of note, exercise had a positive impact on symptoms in 65 patients (52.4%); however, 70 patients (56.5%) reported that physical exertion had a negative impact on their symptoms. Stress and sleep deprivation were reported to worsen symptoms in 92 (74.2%) and 78 patients (62.9%), respectively.

## 4. Discussion

The results from the PRO-AOSD survey provide a direct comparison of patient- and physician-reported outcomes, highlighting areas of consistency and discrepancy between the interpretations of AOSD disease activity. The diagnostic delay of AOSD is a previously reported issue [4], which was confirmed by our study. This cohort experienced a mean delay in AOSD diagnosis of more than 2 years following the first onset of symptoms, which is longer than previously reported [4,18]. A polyphasic phenotype can lead to a delay in diagnosis, as patients are often only diagnosed after several disease recurrences [19]; nearly half of the patients in this study were recorded as having polyphasic AOSD. Previous research has reported that a delay in diagnosis is associated with the development of chronic disease [20]; in this study, more than a third of patients had chronic disease as reported by their physician. The delay in diagnosis emphasizes the necessity of a high degree of suspicion for AOSD at clinical presentation for timely diagnosis and treatment.

Assessments of current pain were similar between patients and physicians, but patients reported significantly greater disease activity. Most patients reported that they felt that their current medication was improving their symptoms, which is reflected in the low mean score for current symptoms. Around two-thirds of patients were receiving a biologic at the time, of whom eight out of every ten patients reported an improvement in symptoms. Multiple studies have shown an improvement in patients’ symptoms if biologic treatment is initiated as early as possible after diagnosis is confirmed, emphasizing the ‘window of opportunity’ for the treatment of AOSD [6,10,11,21,22]. Many patients and physicians reported prior treatment with antibiotics, which are not suitable treatments for AOSD [23,24]; however, current antibiotic use was reported by 1.6% of patients and 1.0% of physicians, which likely results from misdiagnosis of an infection before the AOSD diagnosis was confirmed. Considering that infections can trigger AOSD flares, physicians may often prescribe antibiotics to control the infection. Treatment with glucocorticoids was not included in the surveys.

Despite the low mean score for current symptoms reported by patients, about one-third felt that their disease had worsened in the past year. Of these patients, most of their physicians reported an improvement in their health. This is not surprising when looking at the differences in reported symptoms; patients reported a significantly greater frequency of many symptoms, compared with the frequency reported by their physician, including back pain, fatigue and weakness, and joint inflammation. Similar to this study, multiple studies in rheumatoid arthritis have shown a significant and heterogeneous degree of discrepancy between patients and physicians regarding disease activity. It has been reported that patients often focus on pain and fatigue, while physicians emphasize objective measures like swollen joints and laboratory markers. Greater patient–physician misalignment has been linked to worse clinical outcomes, lower remission rates, reduced work productivity, and poorer HRQoL [25]. Improved communication and integration of PROs could help bridge these gaps and provide support in optimizing patient care.

Despite treatment(s) being currently prescribed, more than a quarter of patients reported experiencing no pain. The low frequency of fever may indicate low disease activity in these patients, but it is also reflective of the physician survey results, which showed that resolution of fever is the most highly prioritized treatment goal.

The prevalence of skin rash reported by patients and physicians in the present survey was low; skin manifestations are relatively simple to treat, which may help to explain why ‘normalization of skin changes’ was not ranked as a priority by the responding physicians. Cardiac complications, such as pulmonary arterial hypertension in AOSD, can be potentially life-threatening [26]. Large datasets on hypertension in AOSD are limited; however, a large study in rheumatoid arthritis (*n* = 17,760) reported that recent glucocorticoid use (>7.5 mg) was associated with a 17% increased risk of hypertension [27]. Considering the relatively young age of the cohort, the prevalence of symptoms associated with cardiovascular risk appears notably high; future studies are needed to address this concern.

The symptom data demonstrate the diversity of symptoms experienced; notably, some of the symptoms are not specific to AOSD. Although most patients in this cohort considered their AOSD to be sufficiently controlled with their current medication, many patients reported fatigue. Although it may not be a symptom specific to AOSD, it is seemingly common in patients with this disease. Patient representatives involved in the development of the Die Deutsche Gesellschaft für Rheumatologie e.V. (DGRh) 2022 guidelines [5] stressed that fatigue is a high priority for patients with a substantial disease burden.

Even though there is no universally accepted and used assessment tool for measuring AOSD activity, it is generally accepted that increased CRP levels are associated with acute and chronic inflammation and can be used for monitoring disease activity [28,29,30]. Disease activity, as determined by CRP levels, indicated that the majority of patients surveyed had inactive disease. There was a significant correlation between CRP-defined disease activity and physician-reported current pain and global disease, indicating that increased CRP levels are associated with higher pain and global disease activity. A previous publication has similarly reported significantly higher CRP levels in patients with active disease versus inactive disease [31]. Given the widespread availability of CRP level analysis [32], these findings could be transferable into clinical practice to improve the management of patients with AOSD.

The present study had some limitations. The ethnic and geographic diversity of this study is limited and may not be directly applicable to populations in other areas of the world. Some of the corresponding physician and patient questions differ slightly, and it is not clear if symptoms as reported by patients and physicians refer to acute or chronic symptom presentation, making it challenging to observe trends and draw clear conclusions. In addition, our evaluation of patients with active versus inactive disease was characterized by a relatively small sample size due to missing or implausible data, meaning further future evaluations are needed to confirm our findings. Furthermore, there is no universally accepted and used assessment tool for AOSD activity, thus limiting the generalization of the results about patients with active and inactive disease.

Overall, there are clear differences between the reported experiences of patients with AOSD and their physicians’ interpretations of the same outcomes. Despite this, the patient’s disease is often well controlled with their prescribed treatment. The differences between physicians’ and patients’ views may imply that there is a need for improved dialogue during clinic visits, to enable physicians to better understand patients’ views and priorities in terms of their treatment and symptoms. Further studies may be warranted, potentially through the incorporation of similar patient and physician/investigator surveys into clinical trials.

## Figures and Tables

**Table 1:** Baseline demographics.

	Total	Male	Female
	N = 124 (100%)	*n* = 50 (40.3%)	*n* = 74 (59.7%)
Age, years, mean (SD)	45.5 (14.7)	44.8 (13.6)	46 (15.4)
Weight, kg, mean (SD)	79.0 (19.1)	89.4 (16.2)	72.0 (17.7)
Height, cm, mean (SD)	170.8 (9.9)	179.3 (7.2)	165.1 (6.8)
BMI, kg/m^2^, mean (SD)	27.0 (5.8)	27.8 (4.7)	26.4 (6.3)
Smoker, *n* (%)	18 (14.5)	7 (14.0)	11 (14.9)

BMI, body mass index; N/n, number of patients; SD, standard deviation.

**Table 2:** Laboratory test results.

	Serum Ferritin (µg/L)	CRP (mg/L)	ESR (mm/h)	AST (IU/L)	ALT (IU/L)	Leukocyte Count (Gpt/L)
*Valid,* *n (%)*	88 (71.0)	107 (86.3)	77 (62.1)	87 (70.2)	107 (86.3)	89 (71.8)
*Missing,* *n (%)*	36 (29.0)	17 (13.7)	47(37.9)	37 (29.8)	17 (13.7)	35 (28.2)
*Median*	118.5	2.5	11.0	27.0	25.0	6.3
*Mean*	562.2	16.04	16.6	30.1	36.8	7.6
*SD*	1879.1	47.9	21.0	15.3	43.0	4.0
*Minimum*	5.0	0.0	2.0	0.0	0.0	3.1
*Maximum*	16,000.0	300.0	100.0	91.0	385.0	28.0

ALT, alanine aminotransferase; AST, aspartate aminotransferase; CRP, C-reactive protein; ESR, erythrocyte sedimentation rate; SD, standard deviation.

**Table 3:** Impact of external factors on disease improvement, as reported by patients.

Please Rate Whether You Have Noticed Any Influence of the Following Factors on Your Symptoms (N = 124)	Positive Influence, Noticeably Improves My Disease Symptoms, *n*, (%)	Negative Influence, Worsens Disease Symptoms, *n*, (%)	No Influence or Does Not Apply, *n*, (%)
More exercise or sport	65 (52.4)	18 (14.5)	41 (33.1)
Fruit/vegetables	39 (31.5)	1 (0.8)	84 (67.7)
Mediterranean diet	32 (25.8)	0 (0.0)	92 (74.2)
Vegetarian/vegan diet	25 (20.2)	2 (1.6)	97 (78.2)
Coffee/tea	13 (10.5)	6 (4.8)	105 (84.7)
Smoke less	10 (8.1)	3 (2.4)	111 (89.5)
Physical exertion	8 (6.5)	70 (56.5)	46 (37.1)
Fruit juices	7 (5.6)	10 (8.1)	107 (86.3)
Stress	4 (3.2)	92 (74.2)	28 (22.6)
Infections (e.g., colds)	3 (2.4)	64 (51.6)	57 (46.0)
Seasonal cold	3 (2.4)	68 (54.8)	53 (42.7)
Sleep deprivation	1 (0.8)	78 (62.9)	45 (36.3)
Increased meat consumption (e.g., daily sausage and meat)	1 (0.8)	30 (24.2)	93 (75.0)
Fast food/finished products	1 (0.8)	29 (23.4)	94 (75.8)
Sugary drinks (lemonade/cola)	1 (0.8)	22 (17.7)	101 (81.5)
Alcohol consumption	0 (0.0)	31 (25.0)	93 (75.0)
Hormonal fluctuations/monthly cycle	0 (0.0)	23 (18.5)	101 (81.5)

Values in this table have been rounded to one decimal place; accordingly, the sum of the individual components may not total 100%.

## Data Availability

The original contributions presented in this study are included in the article/Supplementary Materials. Further inquiries can be directed to the corresponding author.

## Supplementary Materials

The following supporting information can be downloaded at https://www.mdpi.com/article/10.3390/jcm14197034/s1.

## Abbreviations

The following abbreviations are used in this manuscript:

AID	Autoimmune disease
AOSD	Adult-onset Still’s disease
ALT	Alanine aminotransferase
AST	Aspartate aminotransferase
BMI	Body mass index
CRP	C-reactive protein
DAS28	Disease Activity Score 28
DMARD	Disease-modifying anti-rheumatic drug
ESR	Erythrocyte sedimentation rate
HRQoL	Health-related quality of life
N/n	Number of patients
NSAID	Non-steroidal anti-inflammatory drug
PRO	Patient-reported outcome
PRO-AOSD	Patient-reported outcomes adult-onset Still’s disease
SF-36	Short Form-36
sJIA	Systemic juvenile idiopathic arthritis
SD	Standard deviation
WPAI	Work Productivity and Activity Impairment

## References

[1] De Matteis A., Bindoli S., De Benedetti F., Carmona L., Fautrel B., Mitrovic S. (2024). Systemic juvenile idiopathic arthritis and adult-onset Still’s disease are the same disease: Evidence from systematic reviews and meta-analyses informing the 2023 EULAR/PReS recommendations for the diagnosis and management of Still’s disease. Ann. Rheum. Dis..

[2] Fautrel B., Mitrovic S., De Matteis A., Bindoli S., Antón J., Belot A., Bracaglia C., Constantin T., Dagna L., Di Bartolo A. (2024). EULAR/PReS recommendations for the diagnosis and management of Still’s disease, comprising systemic juvenile idiopathic arthritis and adult-onset Still’s disease. Ann. Rheum. Dis..

[3] Seco T., Cerqueira A., Costa A., Fernandes C., Cotter J. (2020). Adult-Onset Still’s Disease: Typical Presentation, Delayed Diagnosis. Cureus.

[4] Efthimiou P., Kontzias A., Hur P., Rodha K., Ramakrishna G.S., Nakasato P. (2021). Adult-onset Still’s disease in focus: Clinical manifestations, diagnosis, treatment, and unmet needs in the era of targeted therapies. Semin. Arthritis Rheum..

[5] Vordenbaumen S., Feist E., Rech J., Fleck M., Blank N., Haas J.P., Kotter I., Krusche M., Chehab G., Hoyer B. (2023). Diagnosis and treatment of adult-onset Still’s disease: A concise summary of the German society of rheumatology S2 guideline. Z. Rheumatol..

[6] Giacomelli R., Caporali R., Ciccia F., Colafrancesco S., Dagna L., Govoni M., Iannone F., Leccese P., Montecucco C., Pappagallo G. (2023). Expert consensus on the treatment of patients with adult-onset still’s disease with the goal of achieving an early and long-term remission. Autoimmun. Rev..

[7] Feist E., Mitrovic S., Fautrel B. (2018). Mechanisms, biomarkers and targets for adult-onset Still’s disease. Nat. Rev. Rheumatol..

[8] Mitrovic S., Fautrel B. (2021). Clinical Phenotypes of Adult-Onset Still’s Disease: New Insights from Pathophysiology and Literature Findings. J. Clin. Med..

[9] Fautrel B., Patterson J., Bowe C., Arber M., Glanville J., Mealing S., Canon-Garcia V., Fagerhed L., Rabijns H., Giacomelli R. (2023). Systematic review on the use of biologics in adult-onset still’s disease. Semin. Arthritis Rheum..

[10] Vitale A., Caggiano V., Maggio M.C., Lopalco G., Emmi G., Sota J., La Torre F., Ruscitti P., Bartoloni E., Conti G. (2022). Canakinumab as first-line biological therapy in Still’s disease and differences between the systemic and the chronic-articular courses: Real-life experience from the international AIDA registry. Front. Med..

[11] Fautrel B., Mitrovic S., De Matteis A., Bindoli S., Anton J., Belot A., Bracaglia C., Constantin T., Dagna L., de Bartolo A. (2023). EULAR/PreS Recommendations for the Diagnosis and Management of Systemic Juvenile Idiopathic Arthritis (sJIA) and Adult Onset Still’s Disease (AOSD) [abstract 0761]. Arthritis Rheumatol..

[12] Park E.H., Strand V., Oh Y.J., Song Y.W., Lee E.B. (2019). Health-related quality of life in systemic sclerosis compared with other rheumatic diseases: A cross-sectional study. Arthritis Res. Ther..

[13] Salaffi F., Di Carlo M., Carotti M., Farah S., Ciapetti A., Gutierrez M. (2019). The impact of different rheumatic diseases on health-related quality of life: A comparison with a selected sample of healthy individuals using SF-36 questionnaire, EQ-5D and SF-6D utility values. Acta Biomed..

[14] Ruscitti P., Rozza G., Di Muzio C., Biaggi A., Iacono D., Pantano I., Iagnocco A., Giacomelli R., Cipriani P., Ciccia F. (2022). Assessment of health-related quality of life in patients with adult onset Still disease: Results from a multicentre cross-sectional study. Medicine.

[15] Doumen M., De Cock D., Pazmino S., Bertrand D., Joly J., Westhovens R., Verschueren P. (2021). Treatment response and several patient-reported outcomes are early determinants of future self-efficacy in rheumatoid arthritis. Arthritis Res. Ther..

[16] Fautrel B., Alten R., Kirkham B., de la Torre I., Durand F., Barry J., Holzkaemper T., Fakhouri W., Taylor P.C. (2018). Call for action: How to improve use of patient-reported outcomes to guide clinical decision making in rheumatoid arthritis. Rheumatol. Int..

[17] Strand V., Schiff M., Tundia N., Friedman A., Meerwein S., Pangan A., Ganguli A., Fuldeore M., Song Y., Pope J. (2019). Effects of upadacitinib on patient-reported outcomes: Results from SELECT-BEYOND, a phase 3 randomized trial in patients with rheumatoid arthritis and inadequate responses to biologic disease-modifying antirheumatic drugs. Arthritis Res. Ther..

[18] Chuamanochan M., Weller K., Feist E., Kallinich T., Maurer M., Kummerle-Deschner J., Krause K. (2019). State of care for patients with systemic autoinflammatory diseases—Results of a tertiary care survey. World Allergy Organ. J..

[19] Franchini S., Dagna L., Salvo F., Aiello P., Baldissera E., Sabbadini M.G. (2010). Adult onset Still’s disease: Clinical presentation in a large cohort of Italian patients. Clin. Exp. Rheumatol..

[20] Kalyoncu U., Solmaz D., Emmungil H., Yazici A., Kasifoglu T., Kimyon G., Balkarli A., Bes C., Ozmen M., Alibaz-Oner F. (2016). Response rate of initial conventional treatments, disease course, and related factors of patients with adult-onset Still’s disease: Data from a large multicenter cohort. J. Autoimmun..

[21] Çolak S., Tekgöz E., Mammadov M., Çınar M., Yılmaz S. (2022). Biological treatment in resistant adult-onset Still’s disease: A single-center, retrospective cohort study. Arch. Rheumatol..

[22] Pardeo M., Rossi M.N., Pires Marafon D., Sacco E., Bracaglia C., Passarelli C., Caiello I., Marucci G., Insalaco A., Perrone C. (2021). Early Treatment and IL1RN Single-Nucleotide Polymorphisms Affect Response to Anakinra in Systemic Juvenile Idiopathic Arthritis. Arthritis Rheumatol..

[23] Owlia M.B., Mehrpoor G. (2009). Adult-onset Still’s disease: A review. Indian J. Med. Sci..

[24] Sola D., Smirne C., Bruggi F., Bottino Sbaratta C., Tamen Njata A.C., Valente G., Pavanelli M.C., Vitetta R., Bellan M., De Paoli L. (2024). Unveiling the Mystery of Adult-Onset Still’s Disease: A Compelling Case Report. Life.

[25] Sacristán J.A., Dilla T., Díaz-Cerezo S., Gabás-Rivera C., Aceituno S., Lizán L. (2020). Patient-physician discrepancy in the perception of immune-mediated inflammatory diseases: Rheumatoid arthritis, psoriatic arthritis and psoriasis. A qualitative systematic review of the literature. PLoS ONE.

[26] Narvaez J., Mora-Liminana M., Ros I., Ibanez M., Valldeperas J., Cremer D., Nolla J.M., Juan-Mas A. (2019). Pulmonary arterial hypertension in adult-onset Still’s disease: A case series and systematic review of the literature. Semin. Arthritis Rheum..

[27] Costello R.E., Yimer B.B., Roads P., Jani M., Dixon W.G. (2020). Glucocorticoid use is associated with an increased risk of hypertension. Rheumatology.

[28] Luan Y.-Y., Yao Y.-M. (2018). The Clinical Significance and Potential Role of C-Reactive Protein in Chronic Inflammatory and Neurodegenerative Diseases. Front. Immunol..

[29] Fujita C., Sakurai Y., Yasuda Y., Homma R., Huang C.-L., Fujita M. (2022). mCRP as a Biomarker of Adult-Onset Still’s Disease: Quantification of mCRP by ELISA. Front. Immunol..

[30] Di Benedetto P., Cipriani P., Iacono D., Pantano I., Caso F., Emmi G., Grembiale R.D., Cantatore F.P., Atzeni F., Perosa F. (2020). Ferritin and C-reactive protein are predictive biomarkers of mortality and macrophage activation syndrome in adult onset Still’s disease. Analysis of the multicentre Gruppo Italiano di Ricerca in Reumatologia Clinica e Sperimentale (GIRRCS) cohort. PLoS ONE.

[31] Ulutaş F., Senol H., Cobankara V., Karasu U., Kaymaz S. (2021). Neutrophil-to-Lymphocyte Ratio and Platelet-to-Lymphocyte Ratio in Adult-Onset Still Disease, their Relationship with Baseline Disease Activity and Subsequent Disease Course: A Single-Center Retrospective Cohort Study. J. Clin. Diagn. Res..

[32] Plebani M. (2023). Why C-reactive protein is one of the most requested tests in clinical laboratories?. Clin. Chem. Lab. Med. (CCLM).

[33] DeTora L.M., Toroser D., Sykes A., Vanderlinden C., Plunkett F.J., Lane T., Hanekamp E., Dormer L., DiBiasi F., Bridges D. (2022). Good Publication Practice (GPP) Guidelines for Company-Sponsored Biomedical Research: 2022 Update. Ann. Intern. Med..

