# Supplementary material for: Evaluating the Divide Between Patients’ and Physicians’ Perceptions of Adult-Onset Still’s Disease Cases: Insights from the PRO-AOSD Survey
Source: J Clin Med. 2025 Oct 4;14(19):7034. doi: 10.3390/jcm14197034 (PMC12525019; doi:10.3390/jcm14197034)
Supplement: Supplementary file 1 [file jcm-14-07034-s001.zip › jcm-3871948-supplementary.pdf]

Evaluating the divide between patients' and physicians' perceptions of adult-onset Still's disease cases: insights from the PRO-AOSD survey

Norbert Blank[1,2], Ioana Andreica[3,4], Jürgen Rech[5,6,7], Zekayi Sözen[8], Eugen Feist[9,10*]

[1]University Hospital Heidelberg, Division of Rheumatology, Department of Internal Medicine V, Heidelberg, Germany;

[2]University Hospital Heidelberg, Center for Rare Diseases Heidelberg (ZSEHD), Heidelberg, Germany;

[3]Ruhr-Universität Bochum, Bochum, Germany;

[4]Rheumazentrum Ruhrgebiet, Herne, Germany;

[5]Department of Internal Medicine 3 - Rheumatology and Immunology, Friedrich-Alexander University (FAU) Erlangen-Nürnberg and Universitätsklinikum Erlangen, Erlangen, Germany;

[6]Deutsches Zentrum für Immuntherapie (DZI), Friedrich-Alexander-Universität Erlangen-Nürnberg and Uniklinikum Erlangen, Erlangen, Germany;

[7]Center for Rare Diseases Erlangen (ZSEER), Friedrich-Alexander-Universität Erlangen-Nürnberg and Uniklinikum Erlangen, Erlangen, Germany;

[8]Novartis Pharma AG, Basel, Switzerland;

[9]Helios Fachklinik Vogelsang-Gommern Klinik für Rheumatologie, Department of Rheumatology and Clinical Immunology, Gommern, Germany;

[10]Otto-von-Guericke Universität Magdeburg, Experimental Rheumatology, Magdeburg, Germany.

*Correspondence: eugen.feist@helios-gesundheit.de

CONFIDENTIAL

CONFIDENTIAL

Patient survey

Introduction

Dear Patient,

Thank you for deciding to participate in this scientific survey, which was conducted by Mondosano GmbH, Henny-Jahnn-Weg 41-45, D-22085 Hamburg, Germany, on behalf of Novartis Pharma GmbH, Roonstraße 25, D-90429 Nuremberg.

For more information about the purpose and procedure of this survey, please contact your doctor and refer to the patient information provided by your doctor.

Before you can start the survey, we would like to ask you to confirm that you have been informed about the survey by your doctor and have signed the patient information and consent form (button for confirmation).

Part I - Demographic data

1. **How old are you?** (in years)

2. **Please enter your gender**

Male	
Female	
Diverse	

3. **How much do you weigh? (in kg)**

Weight between 30 kg and 400 kg	

4. **How tall are you? (indicated in cm)**

Size between 60 cm and 240 cm	

5. **Are you a current smoker?**

Yes/No	

<u>Part II - Questions about the symptoms and physical impairments caused by AOSD</u>

Please note that for reasons of feasibility, we indicate your disease as "adult-onset Still's disease" with the common abbreviation "AOSD" in the questionnaire.

6. **Please assess your current pain:**

(The larger the number entered, the greater the current pain)

Currently, I don't have any pain	0 1 2 3 4 5 6 7 8 9 10	At the moment, I have very strong pain

7. **Please assess your pain over the last 4 weeks:**

(The larger the number entered, the greater the pain felt in the last 4 weeks)

In the last 4 weeks, I had no pain	0 1 2 3 4 5 6 7 8 9 10	In the last 4 weeks, I had very severe pain

8. **Please assess the current symptoms of illness:**

(The larger the number entered, the greater the current symptoms of illness)

Currently, I have no disease complaints	0 1 2 3 4 5 6 7 8 9 10	Currently, I have very severe symptoms of illness

9. **Please estimate the average disease activity of the last 4 weeks:**

(The larger the number entered, the more symptoms in the last 4 weeks)

In the last 4 weeks I had no illness complaints	0 1 2 3 4 5 6 7 8 9 10	In the last 4 weeks, I had very severe symptoms of illness

10. **Do you currently have painful joints?**

Yes, I have painful joints / No, I don't

11. **Do you currently have swollen joints?**

Yes, I have swollen joints / No, I don't

CONFIDENTIAL

_12._ What symptoms do you suffer from today?

	Today	I don't know	I do not suffer from this complaint
Joint inflammation (arthritis)			
Fever			
Sore throat			
Rash			
Frequent fatigue and powerlessness			
Fatigue			
Numbness of areas of the body			
Frequent eye inflammation, possibly accompanied by sensitivity to light and pain			
Lymph node swelling			
Shortness of breath during physical exertion			
Pain when breathing			
Dry cough or cough			
Swelling of the subcutaneous tissue (so-called oedema)			
Arrhythmias			
Backache			
Diabetes mellitus (diabetes)			
Hypertension			

CONFIDENTIAL

_13._ How many years ago did you notice the first signs of illness (symptoms)?
(estimated in years)

_14._ How many years ago was AOSD diagnosed by a doctor, i.e. when did you know what you had? (estimated in years)

15. **Which specialist made the diagnosis of AOSD?**

Rheumatologist	
Orthopaedist	
Doctor in the clinic or hospital	
General practitioner/family doctor	
Dermatologist	
Gastrointestinal doctor/gastroenterologist	
Ophthalmologist	
Other doctor	

16. **Where or by which doctor(s) were you treated before being diagnosed with AOSD?**

Rheumatologist	
Orthopaedist	
Clinic or hospital doctor	
General practitioner/family doctor	
Dermatologist	
Gastrointestinal doctor/gastroenterologist	
Ophthalmologist	
Healer	
Other doctor	

CONFIDENTIAL

17. **Please evaluate whether you have found an influence of the following factors on your illness symptoms:**

	Positive influence: noticeably improves my illness complaints	Negative influence: worsens my illness symptoms	No influence or not applicable
Stress			
Sleep deprivation			
Smoking less			
Infections (eg, colds)			
More exercise or sport			
Hormonal fluctuations/monthly cycle			
Seasonal cold in winter and autumn			
Physical exertion			
Increased meat consumption (e.g. daily sausage and meat)			
Alcohol consumption			
Lemonade/cola			
Fruit juices			
Fruit/vegetables			
Coffee/tea			
Fast food/convenience foods			
Mediterranean diet			
Health-conscious nutrition			
Vegetarian/vegan nutrition			

18. **Have you already taken medication for your AOSD symptoms or are you currently taking medication?**

Yes/No

(If YES question 19 is asked)

CONFIDENTIAL

19. **What medications are you taking or have you previously because of the AOSD taken or administered?**

Drug/medication	Current	Earlier	Never	I don't know
Pain and rheumatism tablets (so-called NSAIDs – non-steroidal anti-inflammatory drugs) and so-called DMARDs (Basic rheumatism therapy)				
Antibiotics				
Biologics, injections (syringes) or infusions				
Skin to be applied prescription treatments (e.g. topical corticosteroids as ointment or cream) due to rash				

20. **Are your symptoms sufficiently improved by the medication you are taking?**

Yes/No

The following questions concern the impact of AOSD on your ability to work and carry out your normal activities.

Please enter the required information or circle the corresponding number.

21. **Has the current situation regarding your professional activity changed due to the corona pandemic?**

Yes/No

(If YES then to question 22)

22. **What changes have the corona pandemic brought about in your work?**

I have been through the corona crisis lost my job	Yes/No
The corona crisis has burdened me in addition to my AOSD disease, which has had a negative impact on my labour productivity impact	Yes/No
Due to the corona crisis, I am afraid of losing my job and have therefore taken sick leave less often, although I should actually recover	Yes/No
I've been trying since the corona crisis to hide my illness from others more than usual	Yes/No

CONFIDENTIAL

The following questions are about the impact of your health problems on your ability to work and manage your daily activities. By health problems, we mean all physical or mental problems or symptoms.

23. **Are you currently employed (paid work)?**

_______NO _______YES

(If YES then continue at 24)

The next questions concern the **last seven days**, except today.

24. **How many hours of work have you missed in the last seven days due to issues related to your AOSD?**

Consider here hours *missed due to AOSD on sick days, delays, going home early, etc.*

(Enter the HOURS)

25. **How many hours of work have you worked in the last seven days for other reasons (e.g. missed holidays, holidays, time to participate in this study)?**

26. **How many hours have you actually worked in the last seven days?**

_______HOURS (If you enter a "0" here, please proceed to question 6.)

27. **How much have your health issues affected your productivity at work over the past seven days?**

Think of days when you were limited in the amount or type of work you could do, days when you did less than you wanted, or days when you didn't do your job as carefully as you usually would have the ability to. If AOSD has had little impact on your work, choose a low number. If the health problems have greatly affected your work, choose a high number.

Please consider only how much your health problems have affected productivity at work.

Health problems had no impact on my work	0 1 2 3 4 5 6 7 8 9 10	Have health problems completely prevented me from working

CONFIDENTIAL

28. **How much have your health problems affected your ability to go about your normal daily activities, excluding work, over the past seven days?**

Under normal activities, we understand the usual activities that you complete, such as housework, shopping, childcare, gymnastics/physical movement, learning, and so on. Think of your health problems at times when you could do limited activities and times in which it is less managed than you wanted. If the health problems only slightly impact your activities choose a low number. If the health problems have strongly impacted your activities choose a high Number.

Just consider how much your health problems have affected your ability to go about your normal daily work, excluding work.

Health problems had no effect on my daily activities	0 1 2 3 4 5 6 7 8 9 10	Health problems have completely affected me and hindered my daily activities

29. **How many days have you been unable to work due to AOSD <u>in the last 12 months</u>?**

Doesn't apply to me	
Less than 1 week	
1 week to 4 weeks	
More than 1 month but less than ≤3 months	
More than 3 months to 6 months	
More than 6 months	
I don't know	

30. **How many days <u>in the last 12 months</u> have you felt too sick to work and still went to work?**

Doesn't apply to me	
Less than 1 week	
1 week to 4 weeks	
More than 1 month to 3 months	
More than 3 months to 6 months	
More than 6 months	
I don't know	

CONFIDENTIAL

31. **How has AOSD affected your professional activity?**

Did you have to limit your career plans due to the AOSD restrictions? abandon?
Yes/No

Have you ever had sick pay or sickness benefit because of your AOSD disease? Daily sickness benefit (i.e. was there an incapacity for work for more than 6 weeks)?
Yes/No

Did you have to give up your profession due to the restrictions imposed by AOSD?
Yes/No

Have you ever had a vocational rehabilitation measure in the company (e.g. a retraining measure)?
Yes/No

Part IV.

The following questions concern the quality of life.
Due to the **corona pandemic**, certain areas of life have changed significantly. In this section we would like to learn how to assess your quality of life, especially as an AOSD patient.

32. **How has the corona pandemic affected your life as a patient with AOSD?**

The corona pandemic burdens me more than other healthy people, especially as an AOSD patient, and additionally limits my quality of life (Yes/No)

I am more afraid than my friends / acquaintances to get infected with the corona virus due to my AOSD disease (Yes/No)

As an AOSD patient, I am at a higher risk of becoming seriously ill with Covid-19 (Yes/No) I feel uncomfortable with doctor and hospital visits and am afraid to deal with the infecting coronavirus (Yes/no)

I am concerned that the medication I am receiving for my AOSD disease could weaken my immune system and thus have a higher risk of contracting Covid-19 (corona disease) (Yes/No)

If YES:

In the past, I have discontinued my medication for the treatment of AOSD for this reason (Yes/No)
(Please talk to your doctor about your fears on this topic)

33. **If you think about the time before the corona pandemic, how has AOSD already affected your life at that time?**

I've met with friends less often (Yes, agrees/No, disagree)

I went partying less often (Yes, true/No, not true)

Due to the illness, I have already stopped my sports activities for the most part (Yes, applies/No, not true)

I have hardly taken a vacation (Yes, true/No, not true)

I have tried different diets during the course of the disease (Yes, agrees/No, not true)

For a long time I have avoided physical proximity to friends (Yes, applies/No, that's not true)

CONFIDENTIAL

Here are general questions about your health and quality of life.

34. **Has AOSD affected your sleep in the last 4 weeks?**

Yes/No

35. **How would you describe the quality of your sleep?**

Very good/Pretty good/Pretty bad/Very bad

Quality of Life Questions: SF-36 Score

36. **How would you describe your state of health in general?**

Excellent/Very good/Good/Less good/Poor

37. **<u>Compared to last year</u>, how would you describe your <u>current</u> state of health?**

Currently much better than a year ago	Currently slightly better than a year ago	About the same as a year ago	Currently slightly worse than a year ago	Currently a lot worse than a year ago

38. **The following questions describe activities that you may do on a normal day.**

Are you limited by your current state of health during strenuous activities, eg, run fast, lift heavy objects, do strenuous sports? If so, how strong?

0 and, strongly restricted	1 and, somewhat restricted	2 no, not restricted at all

(possible answers for the following sub-questions)

If you are limited by your current state of health in moderate work, eg, moving a table, vacuuming, bowling, playing golf? If so, how strong?

Are you limited by your current state of health to lift or carry shopping bags? If so, how strong?

Are you limited by your current state of health to climb several landings? If so, how strong?

Are you limited by your current state of health to climb a landing? If so, how strong?

Are you limited by your current state of health to bend, kneel, or bend? If so, how strong?

Are you limited by your current state of health to walk more than a kilometre? If so, how strong?

Are you limited by your current state of health to walk several hundred metres? If so, how strong?

Are you limited by your current state of health to walk one hundred metres? If so, how strong?

Are you limited by your current state of health to bathe or dress? If so, how strong?

CONFIDENTIAL

39. **How often <u>in the past 4 weeks</u> have you had any difficulties at work or other everyday activities at work or at home due to your physical health?**

(possible answers)

Always (1)	Most (2)	Sometimes (3)	Rare (4)	Never (5)

<u>Due to my physical health</u>, I could not work as long <u>as</u> usual

<u>Because of my physical health</u>, I <u>did less</u> than I wanted

<u>Because of my physical health</u>, I could only do <u>certain things</u>

<u>Due to my physical health</u>, I had <u>difficulties</u> in carrying out my activities (eg, I had to make a special effort)

<u>Due to mental problems</u> (eg, feeling down or anxious) I couldn't work as <u>long</u> as usual

<u>Due to mental problems</u> (eg, feeling down or anxious) I did <u>less</u> than I wanted

<u>Due to mental problems</u> (eg, feeling down or anxious) I couldn't work as carefully as usual

40. **How often <u>in the past 4 weeks</u> have you had any difficulties at work or other everyday activities at work or at home due to mental problems (eg, because you felt down or anxious)?**

Always (1)	Most (2)	Sometimes (3)	Rare (4)	Never (5)

a. I couldn't work as long as usual.............. 1 .... 2 .... 3............ 4...........5

b. I did less than I wanted .... 1 .... 2 .... 3 .... 4 5

c. I couldn't work as carefully as usual........... 1 .... 2 .... 3 .... 4...........5

41. **How much has your physical health or mental problems affected your normal contact with family, friends, neighbours, or acquaintances <u>in the past 4 weeks</u>?**

Not at all	Somewhat	Moderate	Quite	Very much

42. **How severe was your <u>pain</u> in the <u>past 4 weeks</u>?**

No pain	Very light	Light	Moderate	Strong	Very strong

43. **To what extent has the pain prevented you from carrying out your daily activities at home and at work in the past 4 weeks?**

Not at all	Somewhat	Moderate	Quite	Very much

CONFIDENTIAL

44. **These questions are about how you feel and how you've felt over the <u>past 4 weeks</u>. Please choose the answer that best suits your condition.**

Always (1)	Most (2)	Sometimes (3)	Rare (4)	Never (5)

How many times have you been in the past 4 weeks:

Full of life?

Very nervous?

So depressed that nothing could cheer you up?

Calm and serene?

Full of energy?

Discouraged and sad?

Exhausted?

Happy?

Tired?

45. **How often have your physical health or mental problems affected your contact with other people (visits to friends, relatives, etc.) in the past 4 weeks?**

Always (1)	Most (2)	Sometimes (3)	Rare (4)	Never (5)

46. **To what extent does each of the following statements apply to you?**

Entirely true (1)	Applies to a large extent	Don't know (3)	Strongly disagree	Not applicable at all

I seem to get sick a little easier than others

I am just as healthy as other people

I know I expect my health to deteriorate

My health is excellent

47. **Do you feel well informed about your AOSD condition?** (Yes/No)

48. **How do you find out about your illness?**

(Google, social media, doctor/medical staff, pharmacy, patient events, patient groups, journals, acquaintance/family exchange, others)

CONFIDENTIAL

Physician survey

Online questionnaire "AOSD survey"

Introduction

Dear attending physician,

Thank you for deciding to participate in this survey, which was conducted by Mondosano GmbH, Hans-Henny-Jahnn-Weg 41-45, 22085 Hamburg (hereinafter referred to as "**Mondosano**") on behalf of Novartis Pharma GmbH, Roonstraße 25, D-90429 Nuremberg.

Questions about the symptoms and physical impairments caused by AOSD

1. **Please enter your 5-digit ID in the number field**
2. **Now please enter the 5-digit ID of your patient in the number field**
3. **How do you assess the course of AOSD so far? (multiple answers are possible)**
    - Chronic course
    - Monophasic course (so far)
    - Poly- or multi-phase course
    - Articular AOSD with joint involvement
    - Systemic AOSD without joint involvement
    - Over time, AOSD in this patient has progressed from systemic to articular, deteriorated

(Condition: If not "Articular AOSD with joint involvement", skip question X)

4. **Please estimate the tendency of the disease course of the patient to date. (multiple answers are possible)**
    - In the course of time, the overall state of health of my patient has rather improved
    - In the course I see neither an improvement nor a deterioration in the global/overall state of health of my patient
    - In the course of time, the overall state of health of my patient deteriorates
    - I can't estimate this yet due to the short treatment time
5. **Please estimate the patient's current global/total disease activity. (scale 0–10, selection of value by adding the corresponding number)**

Currently symptom-free	0	1	2	3	4	5	6	7	8	9	10	Very strong disease symptoms

6. **Can you assess the patient's current pain?**
    - Yes
    - No

(Condition: If no, skip next question)

CONFIDENTIAL

7. Please estimate the patient's current pain.

No pain	0 1 2 3 4 5 6 7 8 9 10	Very strong intolerable pain

8. Do you have the laboratory results for the serum ferritin value? (Expressed in units: µg/L)

- Yes
- No

(Condition: If no, skip next question)

9. Please indicate the serum ferritin value. (Expressed in units: µg/L. Maximum value: 20,000)

10. Do you have the laboratory results for the CRP value? (Expressed in units: mg/L)

- Yes
- No

(Condition: If no, skip next question)

11. Please enter the CRP value. (Expressed in units: mg/L. Maximum value: 300)

12. Do you have the laboratory results for the ESR value? (Expressed in units: mm/h)

- Yes
- No

(Condition: If no, skip next question)

13. Please enter the ESR value. (Expressed in unit: mm/h. Maximum value: 120)

14. Do you have the laboratory results for the transaminase (AST/ALT) value? (Expressed in units: IU/L)

- Yes
- No

(Condition: If no, skip next question)

CONFIDENTIAL

15. **Please indicate the laboratory results for the transaminase value. (Expressed in IU/L)**

16. **Do you have the laboratory results for the leucocytosis value? (indicated in Gpt/L)**

- Yes
- No

17. **Please indicate the laboratory results for the leucocytosis value. (Indicated in Gpt/L. Maximum value: 30)**

18. **Are the patient's joints affected by swelling?**

- Yes
- No

19. **Please indicate the positions of the currently swollen joints.**

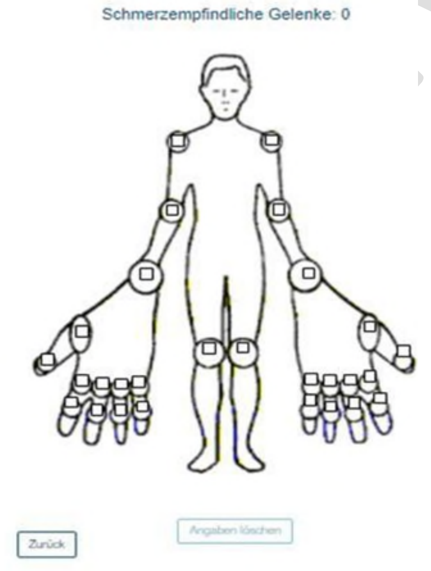

20. **Are the patient's joints affected by pain?**

- Yes
- No

21. **Please indicate the position of the joints that are currently sensitive**

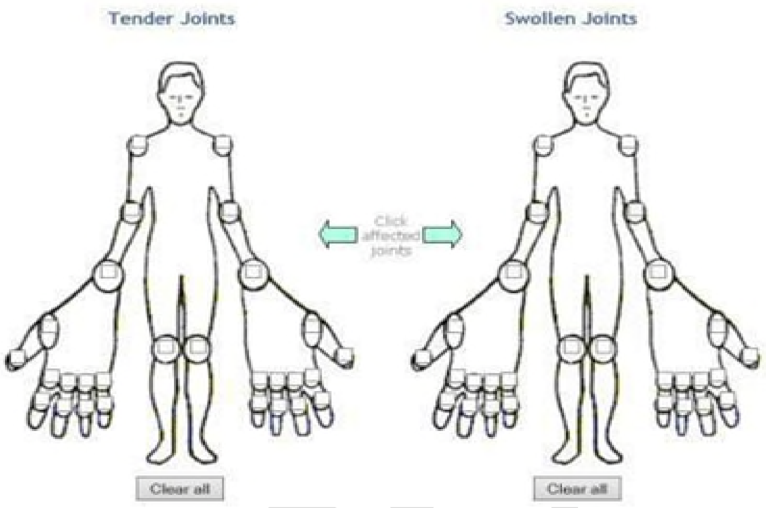

22. **Does the patient currently have concomitant symptoms?**

- Yes
- No

(Condition: If no, skip next question)

23. **What accompanying symptoms do the patient currently have? (Multiple answers possible)**

	Yes	No	I don't know
Joint inflammation (arthritis)			
Fever			
Sore throat			
Rash			
Splenomegaly			
Liver (impairment)			
Pleuritis			
Pericarditis			
Pneumonia			
Lymphadenopathy			
Abdominal pain			

CONFIDENTIAL

Renal failure			
Myalgia			
Weight loss			
Myocarditis			
Frequent fatigue and powerlessness			
Fatigue			
Numbness			
Frequent eye infections may be accompanied by sensitivity to light and pain			
Shortness of breath during physical exertion			
Pain when breathing			
Dry cough or cough			
Swelling of the subcutaneous tissue (oedema)			
Arrhythmias			
Backache			
Other			

24. Did the patient have macrophage activation syndrome (MAS) in the past?

- Yes
- No

25. Is your patient currently receiving medication to treat AOSD?

- Yes
- No

(Condition: If no, skip next question)

26. Which drug group is prescribed to patients?

Drug	Current	Earlier	Never
Non-steroidal anti-inflammatory drugs (NSAIDs)			
Antibiotics			
DMARDs (Basic therapeutics)			
Biologics			
Prescription treatments to be applied to the skin (eg, topical corticosteroids as ointment or cream) due to rash			

CONFIDENTIAL

27. Which therapy goals are important to you?

Please move the answers you consider important to the right with the mouse and order them in order of importance.

If none of the goals are important to you, only move this answer to the right.

- No fever
- Normalization of inflammation levels
- Normalization of joint complaints/arthritis
- Long-term therapy with cortisone-free
- Long-term therapy without NSAIDs
- Normalization of skin change
- None of the stated objectives

CONFIDENTIAL
